# Capture, Sampling and Analysis of Biogenic CO_2_ Streams for Methanol Synthesis

**DOI:** 10.3390/membranes16030106

**Published:** 2026-03-17

**Authors:** Evangelia Koliamitra, Vasileios Mitrousis, Tzouliana Kraia, Giorgos Kardaras, Nikoleta Lazaridou, Triantafyllia Grekou, Kyriakos Fotiadis, Dimitrios Koutsonikolas, Akrivi Asimakopoulou, Michael Bampaou, Kyriakos D. Panopoulos

**Affiliations:** Chemical Process and Energy Resources Institute (CPERI), Centre for Research and Technology Hellas (CERTH), 57001 Thessaloniki, Greece

**Keywords:** bio-CO_2_, methanol, marine fuels, membrane gas absorption, CO_2_ capture, gas cleaning

## Abstract

The shipping sector is responsible for a considerable share of global CO_2_ emissions and is under pressure to reduce emissions and adopt carbon-neutral fuels. Among the proposed alternatives, methanol produced from green hydrogen and biogenic CO_2_ represents a promising option. However, the feasibility of its production is significantly influenced by the composition and variability of the bio-CO_2_ feedstock, which can negatively impact the complete value chain. To address these challenges, sampling campaigns were carried out at actual bio-CO_2_-emitting sites, namely biogas and biomass combustion facilities, to characterize the impurity profiles and determine the appropriate conditioning requirements. A novel membrane gas absorption system with a Diethanolamine solution was deployed directly in the field to capture, as well as purify to a certain extent, the CO_2_ stream. The system demonstrated high efficiency in removing most impurities, achieving high CO_2_ capture rates and impurity reduction close to 90%. However, residual chlorine species were detected in the CO_2_ streams from biogas plants, suggesting the need for additional conditioning to meet the purity specifications required for methanol synthesis. Given that the feedstock composition and upstream process conditions could significantly affect the final output and present considerable variations, the implementation of additional cleaning measures is recommended before synthesis.

## 1. Introduction

The maritime sector accounts for approximately 3% of global greenhouse gas emissions, primarily due to its reliance on fossil-based fuels, such as Marine Diesel Oil and Heavy Fuel Oil. According to the IMO, CO_2_-equivalent emissions increased from 794 Mt in 2012 to over 1076 Mt in 2018 and are projected to exceed 1 Gt annually by 2050 without mitigation measures [1]. Given the projected emissions growth, maritime decarbonization has become an urgent global priority with different solutions being investigated, such as adoption of alternative fuels, on-board carbon capture, energy-efficiency and operational improvements [2]. Among the various options, the adoption of alternative marine fuels and particularly methanol stands out due to its combustion properties, compatibility with existing infrastructure, and liquid state under ambient conditions, which facilitates storage and handling procedures [3]. MeOH production pathways vary, with increasing focus on e-methanol, which can be synthesized from different CO_2_ sources and green hydrogen [4,5]. In most practical configurations, the required CO_2_ is captured from industrial point sources where relatively concentrated streams are available, rather than directly from dilute emissions sources. The composition and purity of the captured CO_2_ stream are critical parameters that influence downstream synthesis performance and overall process feasibility. While the technology is commercially viable, its scalability remains constrained by issues such as increased costs and feedstock availability [6].

The origin of both the hydrogen and carbon feedstock for the synthesis will affect, to a large extent, the environmental and economic performance of the methanol synthesis process [7]. Bio-CO_2_ refers to CO_2_ emitted from industrial facilities that use biomass in the upstream production processes. Key sectors of biogenic CO_2_ include biomass-based facilities, such as biogas/biomethane plants, biomass combustion units, pulp and paper industries, bioethanol facilities, and food and beverage plants [8]. The use of biogenic, rather than fossil-derived CO_2_, is linked to a neutral carbon cycle and does therefore not induce additional stress to the atmospheric carbon cycle. Biomass feedstocks can be utilized upstream through different conversion pathways, resulting in downstream CO_2_ streams with heterogeneous compositions and impurity profiles that may also exhibit seasonal variability [9]. The integration of such biogenic CO_2_ streams into the methanol synthesis value chain poses several technical challenges. The wide spatial distribution of facilities could complicate logistics, whereas the significant variation in plant sizes poses an additional challenge for achieving economies of scale, particularly in smaller facilities such as biogas/biomethane plants with low specific emissions. Furthermore, the diversity of upstream processes and the variability in feedstock composition contribute to the presence of trace impurities, which can affect CO_2_ transport and downstream catalytic performance. In addition, fluctuations in CO_2_ purity and operating conditions (e.g., pressure, moisture content, temperature) further complicate its direct utilization, necessitating dedicated gas cleaning measures. A thorough characterization of each CO_2_ source is therefore essential to ensure stable and long-term operation, irrespective of seasonal fluctuations [10].

CO_2_ captured from different facilities may contain a range of trace contaminants. Their presence and concentrations are influenced by several factors, including the type of feedstock, upstream process conditions, the employed capture technology and downstream purification steps. Common contaminants include oxygen, nitrogen, sulfur compounds (SO_x_, H_2_S), halogens, ammonia, siloxanes, heavy metals and particulates. While not all components pose equal risk, some can severely affect the overall value chain, where even trace amounts can impact catalyst performance, transportation efficiency and system integrity [11]. Similar observations have already been reported by the authors in previous studies [4,5], where investigations into the utilization of steelworks off-gases for methanol and methane synthesis emphasized the need for gas cleaning to prevent catalyst poisoning.

For this purpose, impurity-related risks could span across three key stages of the bio-CO_2_-to-methanol value chain:

(i) Pre-capture, where acid gases such as SO_x_ and NO_2_ irreversibly degrade amine-based solvents, forming heat-stable salts and increasing operational costs. Physical impurities like fly ash can also cause fouling and loss of absorption capacity [12];
(ii) CO_2_ transportation, where impurities affect thermophysical properties and corrosion potential. For pipeline and liquid-phase transport (via ship/truck), adherence to ISO standards is essential to ensure system reliability and safety [13,14,15].
(iii) Methanol synthesis, where the presence of sulphur, chlorine, and oxygen can deactivate the catalyst via sintering, poisoning or structural degradation. Solid particles and trace metals also contribute to performance loss.

To enable high-purity CO_2_ recovery on-site, the modular Membrane-based Gas Absorption (MGA) process could offer a promising solution, even for low CO_2_-emitting sites. This system combines the advantages of selective chemical absorption with membrane technology, allowing for efficient and scalable CO_2_ separation. The MGA process is particularly attractive for decentralized applications like biogas plants due to its modularity, low energy demand, and operational flexibility [16]. Apart from capturing the emitted CO_2_, it could also purify to a certain extent the gaseous stream, thus lowering the cleaning requirements before MeOH synthesis.

Although MGA has been widely studied for CO_2_ capture, most existing work relies on model gas mixtures and emphasizes absorption efficiency rather than CO_2_ purification quality under realistic conditions. Limited studies have evaluated MGA performance with real biogenic streams characterized by humidity, trace contaminants, and fluctuating compositions, nor have they explicitly assessed compliance with downstream methanol synthesis specifications. In this work, MGA is validated under representative biogas and combustion exhaust conditions and directly linked to impurity management and fuel-synthesis requirements, thereby providing a deployment-oriented assessment of the technology for decentralized bio-CO_2_ valorization.

Despite the growing interest in biogenic CO_2_, quantitative field data linking source-specific impurity profiles to catalyst tolerance limits and transport specifications remain limited. In particular, the lack of integrated source-specific measurements across capture, transportation, and synthesis-relevant stages limits evidence-based design of gas cleaning strategies. This work addresses this gap by providing a systematic, multi-stage characterization of real biogenic-CO_2_ streams and directly translating measured impurity levels to conditioning requirements for methanol synthesis. To achieve this, targeted sampling campaigns were conducted at prevailing bio-CO_2_-emitting upstream processes (anaerobic digestion and combustion-related) to characterize the emission composition and identify trace contaminants with potential catalytic or operational impacts. The MGA process was applied to capture and enhance CO_2_ quality. The CO_2_ composition is evaluated at multiple stages of the process, prior to capture, post-capture and pre-synthesis, benchmarking different analytical methods for trace-level quantification and thereby providing a comprehensive basis for selecting conditioning technologies.

The novelty of this work lies in the direct field deployment of a modular MGA system at diverse industrial bio-CO_2_ sites, as well as in the analysis of the gaseous stream along the different steps in the chain. Unlike laboratory studies using synthetic gases, this research provides a comprehensive value-chain analysis, tracking impurities from raw feedstock to transport-ready CO_2_. It further establishes MGA as a dual-purpose technology for both high-rate capture and effective gas purification. This work is organized as follows: Section 2 provides the experimental materials and methods, the sampling and analysis procedures, as well as the deployed capture and regeneration unit. Section 3 presents and discusses the obtained results, and Section 4 summarizes the main conclusions of the study and hints for future research.

## 2. Materials and Methods

**Figure 1:**
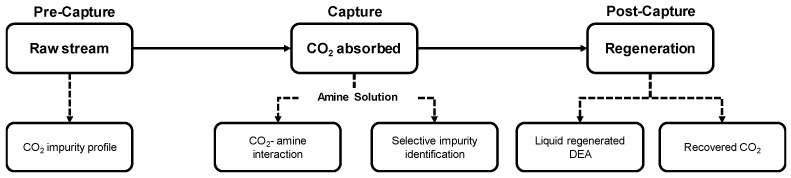
Sequential multi-stage sampling framework. The protocol tracks impurities through raw streams, saturated 2M DEA solvent, recovered CO_2_.

A standardized, multi-point sampling and analysis protocol was applied across the three investigated facilities. Two representative biogenic CO_2_ source types were examined: anaerobic digestion units and biomass combustion. For each case, gas sampling was performed at the outlet of the primary process, upstream of any CO_2_ capture or additional gas cleaning, in order to determine the raw stream composition. The same gas stream was subsequently routed to an on-site membrane gas absorption (MGA) unit operated with an amine-based solvent for capture. After solvent saturation, liquid samples were collected for the quantification of transferred trace impurities. A regeneration step was then carried out to release the captured CO_2_, and the recovered gas was analyzed to determine its final composition. Gas- and liquid-phase analyses at these three defined stages enabled systematic tracking of impurity behavior throughout the capture and purification sequence (Figure 1).

### 2.1. Description of Biogenic CO_2_ Sources

Three industrial-scale biogenic CO_2_ sources in Northern Greece were selected for the sampling campaigns, representing anaerobic digestion (Biogas Plants No.1 and No.2) and biomass combustion (Biomass combustion plant). These facilities provide diverse impurity profiles reflecting typical agricultural and woody biomass feedstocks. All measurements were conducted under steady-state operational conditions at nominal capacity to ensure sample integrity. Sampling points were strategically located downstream of existing gas cleaning systems to characterize the actual emitted streams intended for CO_2_ capture. Technical specifications for each facility are summarized in Table 1.

### 2.2. Bio-CO_2_ Sampling and Analysis Procedures

The sampling strategy was designed to assess variations in CO_2_ content, with two defined points: the direct analysis of the raw stream and the analysis of the MGA-treated stream. Measurements were performed in series for biogas plants and simultaneously under isokinetic conditions for biomass combustion plants. Due to the continuous and stable flue gas velocities in combustion facilities, isokinetic sampling was applied to ensure accurate and representative sampling of both particulate matter and gaseous compounds. In contrast, biogas plants operate under batch or semi-batch regimes, as anaerobic processes typically exhibit retention times of ~60 days. This distinction between continuous and batch operation directly affects both the selection of sampling methodology and the interpretation of analytic results, emphasizing the need to align protocols with facility-specific process dynamics.

#### 2.2.1. Sampling Procedure in Biogas Facilities

**Figure 2:**
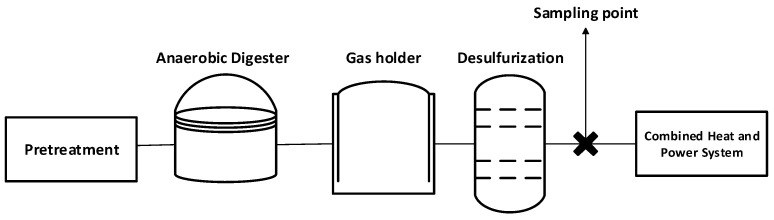
Schematic of the sampling and CO_2_ recovery points at the anaerobic digestion facilities. Points are located downstream of the desulfurization unit and upstream of the CHP unit.

During the sampling campaign, two in-series measurements were conducted: one focusing on the bio-CO_2_ stream analysis and the other on evaluating the membrane-based system application, making sure that the same stream is both analyzed and utilized in the capture unit. The schematic (Figure 2) illustrates a standard biogas process from biomass pretreatment and anaerobic digestion through gas conditioning, including gas holding (buffering) and desulfurization. The sampling point is located downstream of the desulfurization unit and upstream of the CHP unit, allowing the assessment of trace impurities in treated biogas prior to final utilization. The pressure and temperature conditions at the sampling points for the two experiments were 20 bar and 80 °C for the first and 23 bar and 115 °C for the second biogas plant.

The raw gas samples were collected using Tedlar^®^ gas sampling bags (Sigma-Aldrich, St. Louis, MO, USA) and transported for off-site analytical characterization. Particular emphasis was given to sulphur and siloxane compounds, with siloxanes being common impurities contained at significant concentrations in biogas streams, leading to potential fouling and failures in downstream equipment [17,18,19]. Subsequently, the MGA unit was connected for a 2 h operation, where, after solvent saturation, the liquid samples were collected and stored to perform further analysis, both in the amine solvent as well as in the recovered bio-CO_2_ to identify the maximum concentration of impurities in the methanol synthesis reactor inlet. More information on the saturated amine analyses is included in Section 3.2 and in the Supplementary Material.

#### 2.2.2. Sampling Procedure in Biomass Combustion Plant

In this case, a comprehensive analysis of the biomass feedstock was conducted to better understand its composition and potential impact. The detailed characterization focuses on elemental and inorganic constituents relevant to downstream CO_2_ processing. In addition, representative gas samples were collected from the combustion unit following a structured sampling methodology to enable reliable analytical evaluation. Water-tube boilers operate at high temperatures under well-controlled combustion conditions, facilitating efficient ash separation. Biomass ash is typically divided into bottom ash (composed of heavier, less volatile elements) and fly ash, which contains more volatile constituents suspended in the flue gas phase [20]. The volatility of elements enables a preliminary prediction of the flue gas impurity profile, guiding the design of targeted sampling campaigns. These predictions are based on feedstock composition and the impurity tolerance thresholds defined by methanol catalyst specifications [12]. On the day of the sampling, the feedstock consisted of 99% wood and 1% pellet. Biomass feedstock samples were collected and prepared according to ISO 14780-Solid Biofuels-Sample Preparation [21] and quantification of trace elements was conducted using ICP-MS/XRF equipment (7850 ICP-MS, Agilent Technologies Inc., Santa Clara, CA, USA). The elemental composition of biomass feedstock, including carbon, hydrogen, and nitrogen contents, was determined according to the EN15404:2011 standard, which describes instrumental methods for the quantification of total carbon, hydrogen and nitrogen in solid fuels [22]. The focus was on the elemental structure and on key inorganic elements such as alkali metals, chlorine and ash-forming compounds, which are known to influence combustion behavior and the formation of gas-phase impurities [23].

**Figure 3:**
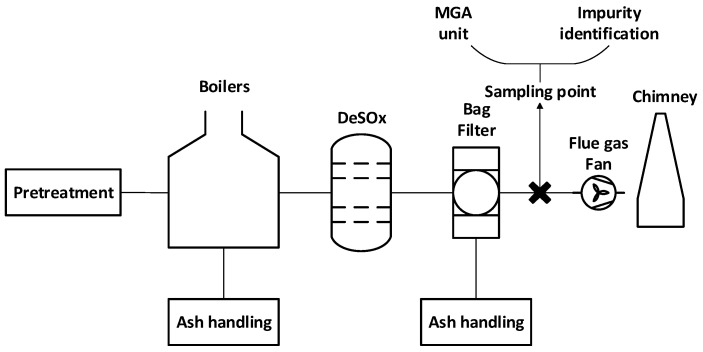
Layout of the sampling and CO_2_ recovery configuration at the biomass combustion plant. Points were installed downstream of the bag filters in a laminar flow zone.

In this sampling campaign, measurements were conducted under isokinetic conditions to ensure representative sampling of the gas stream. Throughout the sampling campaign, one of the two boilers was operational, contributing a power of 12 MW, very close to its nominal value. Under these conditions, steady-state operation was maintained, providing high integrity of the sample collection for impurity identification. The weather conditions proved harsh, with temperatures ranging between approximately 1 and 5 °C. This resulted in increased heat demands in the region and led to the boiler operating at near-nominal values at 600–650 °C. Two sampling points were installed downstream of the filter (Figure 3), where the flow exhibited laminar characteristics. The resulting flue gas exited the facility at 1 bar, 130 °C and with a flow rate of 9.7 L/min (at the pitot entrance). One sample line was used to perform the MGA process, utilizing a similar system as in the biogas campaigns, while the other one was designated for the analysis of the flue gas stream.

**Figure 4:**
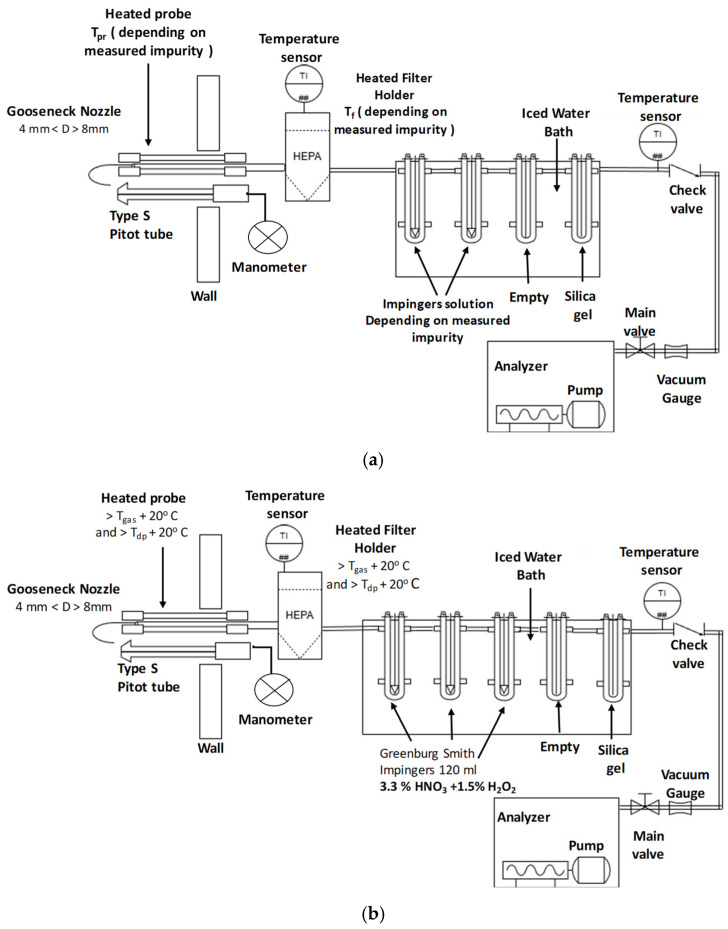
Experimental setup for the analysis of flue gases (**a**) Setup for the identification of halogens (HCl, HF) and mercury (Hg). Impingers solutions: 100 mL chloride-free water (HCl), 100 mL NaOH 0.1 M (HF), Greenburg Smith impingers 100 mL 4% K_2_Cr_2_O_7_ + 20% HNO_3_. T_pr_ = T_f_ = 160 °C for halogens, T_pr_ = T_f_ > T_gas_ + 20 °C. (**b**) Set up for the identification of heavy metals.

To ensure isokinetic sampling, a probe, equipped with a Pitot tube, was positioned at the gas collection point. A filtration system was implemented to separate particulate matter from the flue gases, followed by a series of impingers containing specific absorption solutions. The sampling line was heated up to the entry point of the gas stream into the impinger system to prevent condensation. The number of impingers and the type of reactant solution varied depending on the specific impurity being analyzed (Table 2). In all cases, the final stage of the impinger array consisted of a column filled with silica gel for the removal of residual moisture, preventing potential interference with the analytical process (Figure 4).

The sampling system operated through a pump, controlling the flow rate. At the same time, it measured the pressure drop in the Pitot tube in order to determine the gas velocity and verify the isokinetic conditions. As discussed previously, the impingers contained specific sorbent solutions chosen to selectively capture the target gas species. Inside each impinger, the selected gas reacted with the corresponding solution, while the unreacted gases were passed through and exited the system. To improve the capture efficiency, multiple impingers were used in each measurement, resulting in a higher overall capture rate and minimizing potential losses. After absorption, the resulting liquid mixture containing the dissolved reaction products was collected and subjected to further analytical procedures to determine the concentration of the collected species. The concentrations of O_2_, CO_2_, SO_2_, CO and NO_x_ in the flue gas stream were determined on a dry gas basis, using a portable gas analyzer (ENVEA MIR 9000P, Poissy, France). The analysis of impurities in the carbon dioxide stream aims to identify and quantify the presence of halogens and heavy metals. The campaign duration exceeded 2 h, allowing saturation of the solvent under steady-state operation. Following the completion of absorption, the CO_2_-rich solvent samples were collected and transported to the laboratory for off-site analysis. Consistent with prior procedures, the selection of analytical methods was based on standardized protocols. As for the targeted analysis of saturated amine, the selected analytical methods are the same as those used in biogas sampling campaigns and are described in detail in Section 3.2.

### 2.3. CO_2_ Capture Apparatus and Procedures

**Figure 5:**
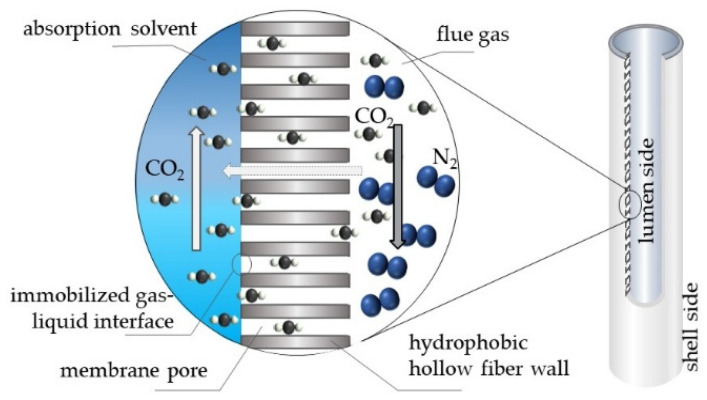
Mechanism of the membrane gas absorption (MGA) process. The diagram illustrates the gas–liquid interface within the hydrophobic hollow fiber pores, where selective chemical absorption occurs [24].

To capture the bio-CO_2_ from each stream, an innovative approach was adopted, namely membrane gas absorption (MGA). It is a hybrid technology for CO_2_ capture that combines the principles of chemical absorption with membrane-based separation. In MGA systems, hollow fiber membrane contactors act as a physical barrier between the gas and liquid phases, allowing efficient CO_2_ transfer without phase mixing [16,24]. Using hydrophobic hollow fiber membranes ensures that the pores remain gas-filled, while maintaining the liquid pressure slightly above the gas pressure, creating a stable interface that enables efficient CO_2_ absorption with minimal mass transfer resistance (Figure 5).

**Figure 6:**
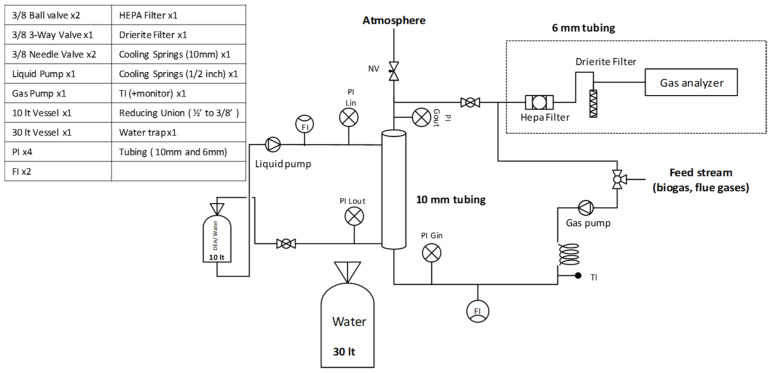
Process flow diagram of the modular MGA capture and analysis unit. The system features the 3M Liqui-Cel^TM^ contactor, Ismatec BVP-Z pumps and the Cubic-Ruiyi Gasboard-3200 analyzer.

Compared to conventional packed columns, MGA offer several advantages: high and constant specific contact area (1500–7000 m^2^/m^3^ vs. 100–800 m^2^/m^3^), flexibility to operate under wide range of gas–liquid ratios without flooding or unloading issues, and the ability to handle diverse CO_2_ concentrations, from industrial flue gases (5–20%) to raw biogas (45–50%). This technology system is compact, modular and easily scalable, enabling predictable performance with lower maintenance costs. The MGA unit consists of two main sections that interact within the membrane module: the liquid phase and the gas phase sections. Both sections are fully equipped with dedicated instrumentation for independent control, operation and analysis. The gas phase section involves the introduction of a CO_2_-rich stream, which is either flue gas or biogas, directed from the plant through a series of valves, pressure and temperature indicators into the lumen side of membranes where the absorption process takes place. Its volumetric flow rate is controlled and monitored using a dedicated gas pump and flow meter. The gas outlet stream is dried through a HEPA and a drierite filter to remove humidity and any particulate matter before entering the gas analyzer and then ventilated into the atmosphere. In the liquid section, an aqueous Diethanolamine (DEA99%, Chem-Lab NV, Zedelgem, Belgium; now AnalytiChem Belgium NV) solution at 2M, prepared in the laboratory, is pumped from the feed tank to the shell side of the membranes in counter-current flow configuration. The CO_2_-rich solvent is then recirculated back into the feed tank. A detailed process flow diagram of the capture apparatus is shown in Figure 6.

The membrane module used was a 2.5 × 8 Liqui-Cel^TM^ 3M EXF membrane contactor (Charlotte, NC, USA, Liqui-Cel^TM^). Detailed technical characteristics of the module are provided in Table 3. Prior to each experimental campaign, the membrane—though not new—was conditioned by overnight drying under a nitrogen (N_2_) atmosphere. The pumps used to deliver the gaseous and liquid streams to the membrane were a Rietschie Thomas 107 series (Rietschle Thomas GmBh, PubChem, Germany) and an Ismatec BVP-Z (Ismatec ISM446B-230V (B-MOUNT) BVP-Z Analog Gear Pump Drive, Glattbrugg, Switzerland), respectively. The gas analyzer was the Cubic-Ruiyi Gasboard-3200 Online Infrared Biogas Analyzer (Hubei Cubic-Ruiyi Instruments Co., Ltd., Wuhan, China, Gasboard-3100 Serial Syngas Analyzer); its detailed technical characteristics can be seen in the Supplementary Material.

The experimental procedure involved a systematic sequence of steps. Initially, the liquid solvent was prepared and loaded into the system. A thorough analysis of the feed gas streams’ composition and total flow measurement was performed. Both the gas and liquid streams were fed into the membrane module. Throughout the process, the liquid phase pressure was carefully monitored and regulated to be slightly higher than the gas phase pressure, preventing any unintended dispersion while minimizing long-term wetting effects. Real-time analysis of the gas effluent’s composition was performed using a gas analyzer. Finally, the effect of various process parameters, such as gas and liquid flow rates and the gas-to-liquid (G/L) ratio, on the overall performance was continuously monitored. The operating conditions recorded during the sampling and system performance evaluation are summarized in Table 4. These parameters reflect the pressure, temperature, and flow rate of both gas and solvent at the MGA feed, providing essential context for understanding system behavior under real operational conditions.

The campaign was considered complete upon full saturation of the amine solution. Subsequently, a comprehensive screening was performed on the saturated amine-CO_2_ solution to quantify trace elements and potential impurities absorbed within the mixture. More specifically, density was measured at 25 °C using a Digital Density Meter (ASTM D42052-18a) (DMA 35, Anton Paar GmBh, Graz, Austria) to identify the increase that occurred since capturing. To further characterize these interactions, Fourier Transform Infrared Spectroscopy (FT-IR) was performed on both saturated and unsaturated solvent samples using a JASCO FT/IR-6700 spectrometer (Jasco Corporation, Tokyo, Japan). The spectral analysis enabled the identification of vibrational modes corresponding to carbamate and carbonate functional groups formed during CO_2_ absorption, as well as potential peaks attributable to impurities present in the CO_2_ stream. The FT-IR spectra were processed using the instrument software, applying water signal correction and spectral smoothing to reduce noise, while no baseline correction was applied. The analysis was intended primarily for qualitative comparison of solvent loading and regeneration states rather than quantitative determination of absorbed species; therefore, peak integration was not performed. This analysis facilitated the estimation of the maximum concentration of impurities potentially present in the recovered CO_2_ stream, assuming full recovery efficiency (more information in the Supplementary Material).

### 2.4. Gas Stripping Process-Solvent Regeneration

A gas stripping unit was employed to generate the solvent batches up to 0.33 L by controlled heating, which facilitated both CO_2_ recovery and solvent regeneration. The apparatus consisted of a heating mantle, boiling flask, and vertical condenser, with batches corresponding to 50–66% of flask volume to ensure efficient heat transfer and controlled evaporation. Thermal desorption was carried out at 95 °C for 1 h. The released gas was withdrawn using a CO_2_Meter diaphragm pump (model PMP-0012, nominal lifetime 10,000 h, maximum flow rate 0.8 L min^−1^, Ormond Beach, FL, USA). No carrier gas was employed, and the stripping relied exclusively on thermal desorption of the dissolved CO_2_. Process temperature was regulated using an integrated control of the heating mantle, whereas gas samples were collected through a three-way valve at the condenser outlet, with leak-tight operation secured by clamps. The heating mantle (ISOLAB Laborgeräte GmBh, with magnetic stirrer, Eschau, Germany) provided precise control up to 450 °C and variable stirring speeds, while tap water was continuously supplied to the condenser as a cooling medium for efficient condensation of solvent vapors.

The experimental procedure involved preparing the gas stripping apparatus, transferring a measured volume of solvent into the boiling flask, and verifying gas-tightness through leak testing. The heating mantle was then activated, with temperature and pressure continuously monitored, while desorbed gases and regenerated solvent samples were collected for analysis. In total, three lab-scale desorption experiments were conducted using saturated solvents from the biogas and biomass combustion facilities. The desorbed CO_2_ stream was collected during each desorption experiment to determine the composition of the released gases, with particular emphasis on CO_2_, hydrocarbons and trace impurities. Analysis was performed using an Agilent 7890B Gas Chromatograph (GC) (Agilent Technologies Inc., Santa Clara, CA, USA) configured with four valves, two thermal conductivity detectors (TCD) and one flame ionization detector (FID), and equipped with HayeSep, Molecular Sieves, DB-1 and HP-AL columns. The FID channel was used for C1–C5 hydrocarbon analysis, while the first TCD channel (He reference gas) was used for permanent gases (CO_2_, CO, O_2_, N_2_ and H_2_S) and the second TCD channel (N_2_ reference gas) for hydrogen detection. The GC system was calibrated using certified calibration gas mixtures containing the target components. The method allowed quantification of permanent gases (CO_2_, CO, N_2_, O_2_, H_2_), light hydrocarbons (C_1_–C_5_) and H_2_S, with detection limits in the range of 0.01 vol% for hydrocarbons,-0.1 vol% for permanent gases, and 0.05 vol% for H_2_S mol%.

### 2.5. Analytic Methods and Equipment

Given that the sampling campaign involved gas–liquid and gas–gas phase measurements, standardized analytical methods were applied throughout the analysis.

Within this framework, analytical methods were applied for the characterization of the flue gas stream. Acid gases (HCl, HF and SO_2_) were quantified by ion chromatography (ISO15713, EN14791 and EN ISO10304) [27,28,29]. Total mercury (Hg) was determined using fluorescence spectrometry (EN ISO17852, EN13211 and ISO12846) [30,31,32]. Heavy metals were quantified by inductively coupled plasma atomic emission spectrometry (ICP-AES) based on EPA Method 200.7.

Analytic methods were also applied to quantify selected inorganic impurities in the amine solutions collected during the sampling campaigns. Specifically, halides and sulphate were quantified by ion chromatography following SMEWW 4500. In addition, sulphides were determined spectrometrically in accordance with ISO10530:2002 [33]. Arsenic was also performed following ASTM D2972-03, employing photometric and atomic absorption techniques [34]. Mercury was analyzed according to ASTM D3223:2000, involving atomic absorption spectroscopy (CVAAS), while antimony was quantified by flame atomic spectroscopy (FAAS), in accordance with ISO15586:2003 [35,36].

As part of the analytical quality assurance procedure, the blank method was systematically applied during the measurements. Blank samples were analyzed under the same experimental and instrumental conditions as the actual samples, in order to account for potential background signals, reagent impurities, or contamination originating from the sampling and analytical setup. The blank values were considered during data evaluation to ensure that the reported concentrations reflect only the contribution of the investigated CO_2_ streams and absorbed species.

Due to the industrial-scale nature of the facilities and operational constraints, full experimental replication was not feasible for all sampling stages. Measurements were conducted under steady-state operating conditions during dedicated sampling campaigns. Reported uncertainties reflect analytical measurement uncertainty rather than experimental repeatability.

## 3. Results

To systematically profile bio-CO_2_ stream contaminants throughout the value chain and evaluate the potential presence of certain impurities in the final CO_2_ stream, analyses were conducted at the (Figure 1): (i) raw streams prior to the capture system, (ii) saturated amine solution, and (iii) recovered bio-CO_2_ after the MGA unit. This multi-step approach enabled the construction of a complete picture of the possible impurities contained in the CO_2_ stream, including their persistence through the various stages of processing. For each of the conducted sampling campaigns, the results are presented in this section and evaluated against established tolerance limits for the methanol synthesis catalyst, as well as specifications relevant to transportation.

### 3.1. Biogas Plants Campaigns Results

#### 3.1.1. Pre-Capture Measurements 

An overview of the results gathered from the average composition of the two biogas campaigns is presented below (Table 5):

The biogas stream analysis from both facilities demonstrated a consistently high methane content, accompanied by low oxygen levels and well-controlled hydrogen sulfide (H_2_S) concentrations. Notably, no hydrogen sulfide was detected at the first plant based on the equipment quantification limits. In contrast, the results from the second biogas plant showed slightly elevated H_2_S concentrations, indicating that downstream processing might be required. Measurement repeatability was better than 1%, supporting the robustness of the reported biogas composition results.

Additional analysis was performed to determine the occurrence of siloxanes in the CO_2_ produced stream. The calibrated data revealed consistent contaminant profiles at both plants, indicating quantities below the quantification limits (more information in the Supplementary Material). The absence or presence of siloxanes in the biogas streams is, among others, attributed to the composition of the feedstock. During periods in which the feedstock is rich in agricultural residues, the siloxane content is typically low [37], as during the two sampling campaigns. Higher siloxane quantities are consistently observed in biogas produced from feedstocks such as municipal solid waste or wastewater treatment sludge, where organosilicon compounds from consumer products (e.g., cosmetics, detergents) introduce siloxanes into the process [37,38]. Although the present results indicate minimal siloxane contamination, such findings should not be generalized, as biogas feedstock composition and process inputs can vary seasonally. Nevertheless, for the utilization of bio-CO_2_ from biogas plants, the potential siloxane presence, detection and subsequent cleaning should be taken into consideration. Targeted removal systems, such as activated carbon adsorption or silica gel scrubbers, could prevent equipment corrosion and catalyst deactivation [39,40].

#### 3.1.2. Measurements in Saturated Amine Solutions

This section presents the analytical results obtained from the examination of the saturated DEA solutions used during CO_2_ capture campaigns at the biogas facilities. The analysis focused both on the physicochemical effects of CO_2_ interaction with the solvent and on the presence of transferred contaminants from the biogas stream. The following subsections present: (i) the physical and spectroscopic evaluation of the solvent, highlighting changes related to CO_2_ absorption and potential impurity-related signals, and (ii) the targeted quantification of inorganic and ionic species, such as halogens, sulfur and heavy metals, using standardized analytical protocols.

**Figure 7:**
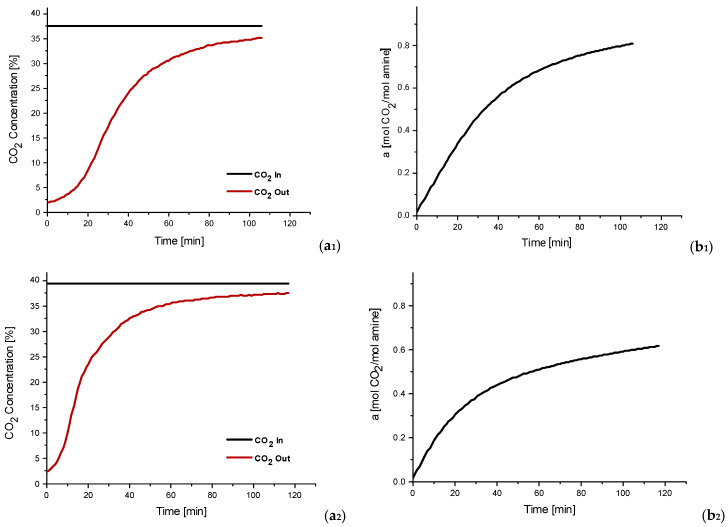
Experimental results of MGA unit during 2-h biogas plants campaigns: (**a_1_**) CO_2_ concentration (vol%) variation in the gaseous stream after MGA with time in Biogas plant No.1, (**b_1_**) Absorption solvent CO_2_ loading (mol CO_2_/mol DEA) in Biogas plant No.1, (**a_2_**) CO_2_ concentration (vol%) variation in the gaseous stream after MGA with time in Biogas plant No.2, (**b_2_**) Absorption solvent CO_2_ loading (mol CO_2_/mol DEA) in Biogas plant No.2.

The experimental results of the MGA campaigns conducted at the biogas plants are presented in Figure 7, which illustrates the temporal evolution of CO_2_ concentration in the gas stream downstream of the MGA unit and the corresponding CO_2_ loading (α) of the absorption solvent. Over the 2-h experimental period, the CO_2_ concentration downstream of the MGA gradually approached the inlet (upstream) concentration, indicating the system’s progression towards solvent saturation. The CO_2_ loading capacity (α) is defined as the molar ratio of absorbed CO_2_ to the total moles of DEA present in the solution. Under standard absorption conditions, aqueous DEA solutions typically exhibit a loading capacity in the range of 0.4–0.5 mol_CO_2__/mol_DEA_, while the theoretical maximum value can reach up to 1 mol_CO_2__/mol_DEA_. CO_2_ loading results obtained during the MGA campaigns confirmed effective performance at both sites. In biogas plant No.1, the 2M DEA solution reached a loading capacity exceeding 0.8 mol_CO_2__/mol_DEA_ after 2 h of operation. In biogas plant No.2, the measured loading surpassed 0.6 mol_CO_2__/mol_DEA_ under comparable conditions. The observed differences likely reflect variations in feed gas composition, temperature, or other process-specific parameters influencing absorption kinetics. In all cases, no detectable solvent was present at the gas outlet side. The experimental campaigns, conducted using the on-site MGA unit, were not designed to provide a detailed investigation of absorption kinetics, including mass-transfer limitations required to fully assess the dynamic performance of the system. As the campaigns consisted of short-duration, single-run measurements, the reported results should be interpreted as representative trends rather than statistically averaged values.

**Figure 8:**
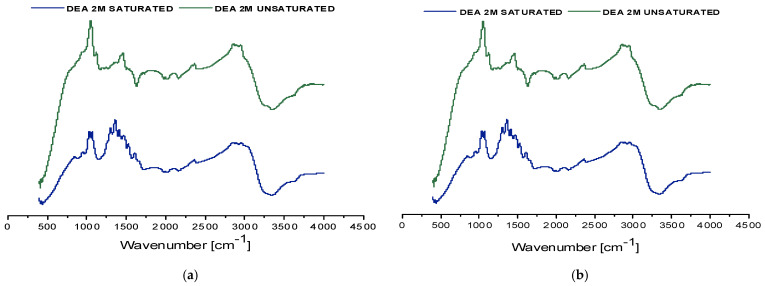
FT-IR spectra (cm^−1^) of saturated and unsaturated 2M DEA samples obtained at the MGA campaigns in the biogas plants. Spectrum (**a**) corresponds to samples from biogas plant No.1, while spectrum (**b**) represents biogas plant No.2.

Density measurements revealed that in both biogas Plant No.1 and Plant No.2, CO_2_ saturation resulted in an increase of about 0.0512 g/mL, confirming efficient CO_2_ absorption. The comparison of the spectra (FT-IR analysis) between unsaturated and saturated samples shows overall similarity for both biogas plants, with minor variations such as the appearance of new absorption bands or shifts in existing peaks (Figure 8). These spectral differences are attributed to the chemical transformations occurring during CO_2_ uptake, including the formation of new bonds and species not present in the unsaturated sample. A detailed assignment of spectral features is provided in the accompanying Table 6. Notably, in the saturated solvent sample from biogas plant No.2 campaign, absorbance bands in the range of approximately 2600–2500 cm^−1^ were observed. These may indicate the presence of HS^−^ species, suggesting the partial absorption of hydrogen sulfide in addition to CO_2_.

To quantify impurities transferred to the solvent during CO_2_ capture, solution composition analysis was performed using certified reference standards, focusing on halogens, sulfur compounds and heavy metals, and, similar to the previous cases, the pure solvent was also analyzed to identify any residual impurities that may have been formed from previous operations. The overall measurement uncertainty of the analytical methods was estimated to be in the range of 10–20%.

Table 7 shows the amount of trace components captured by the amine process expressed as maximum potential concentrations in the purified CO_2_ stream. In practical MGA systems, solvent regeneration is performed in a conventional stripper where CO_2_ is released by heating the CO_2_-rich solvent. Under these conditions, the desorption behavior of co-absorbed trace species differs from that of CO_2_ due to differences in volatility and chemical interactions with the amine solvent, as reported for pilot-scale amine-based CO_2_ capture systems, where impurity and degradation compounds exhibit distinct emission and release behavior during absorber-stripper operation [41]. Consequently, the regeneration efficiency of most impurities is typically lower than that of CO_2_, meaning that only a fraction is expected to be released during stripping, while some may remain dissolved in the solvent. Therefore, the impurity concentrations reported in Table 7 represent conservative upper-bound estimates, and the actual impurity levels reaching the methanol synthesis reactor are expected to be lower under realistic operating conditions. More specifically, the concentration “per CO_2_ absorbed” was calculated assuming a theoretical maximum scenario where 100% of the captured CO_2_ and its associated impurities are recovered during the regeneration step. The corresponding impurity concentrations at the methanol synthesis reactor inlet were calculated on a stoichiometric basis, assuming an ideal CO_2_:H_2_ molar ratio of 1:3. Therefore, the reported values represent conservative upper-bound estimations of impurity concentrations at the reactor inlet. For most impurities, the measured concentrations were significantly below the catalyst tolerance limits provided by the commercial catalyst provider. Overall, chloride removal emerged as an essential conditioning step required for the utilization of bio-CO_2_ in the process. Notably, although the H_2_S concentration in the bio-CO_2_ stream of plant No.2 initially reached 20–30 ppm, it was substantially reduced following treatment with the MGA unit. These results confirm the high efficiency of the membrane system in removing sulphides, thereby reducing the need for extensive H_2_S treatment prior to catalyst exposure. Nevertheless, due to the potential variation in the upstream composition, catalyst protection measures should be taken into account prior to methanol synthesis, such as guard beds for the removal/containment of any potential impurities that might have escaped the previous steps, to allow for a flexible methanol synthesis process that can handle different kinds of bio-CO_2_ feedstocks [4,5].

Among the detected impurities, chlorides were present at significant levels only in CO_2_ derived from biogas, posing a risk to downstream methanol synthesis. This underscores the need for targeted chloride removal. Wet scrubbing, typically using NaOH or Ca(OH)_2_, enables highly efficient removal by converting halogen compounds into water-soluble salts, and is widely applied in spray towers or packed columns [42]. For dry or low-moisture gas streams, dry scrubbing with solid alkalis (e.g., NaOH pellets or NaHCO_3_) offers an effective alternative, although sorbent replacement is required [43]. Absorption onto activated carbon or microporous material is another viable method, with regeneration achieved via heating or inert gas purging [44]. The optimal halogen removal approach depends on gas composition, moisture levels, and downstream purity requirements, with wet scrubbing preferred for moist streams and the dry or adsorption method used in low-humidity or trace-contaminant conditions [42,44,45].

#### 3.1.3. Regenerated CO_2_

**Figure 9:**
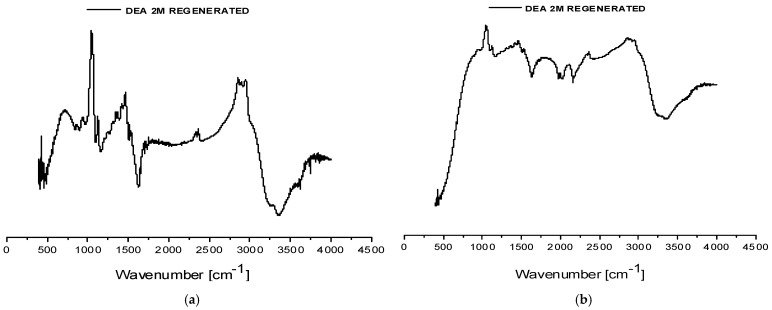
FT-IR spectra (cm^−1^) of the regenerated 2M DEA sample from the campaign in biogas plant No.1 (**a**) and from the campaign in biogas plant No.2 (**b**).

Four samples were collected: two gas-phase samples and two liquid-phase samples consisting of regenerated solvents. The liquid samples were characterized by FT-IR spectroscopy (Figure 9) and density measurements (Table 8) to facilitate comparison with both fresh and saturated solvent states.

The FT-IR spectra of both regenerated solvent samples exhibit identical peak positions to those of the corresponding saturated samples, with variations observed only in peak intensities. This indicates that the regeneration process preserved the chemical bond structure while reducing the concentration of CO_2_-loaded species. The measured densities of the regenerated solvents, shown in Table 8, are slightly higher than those of fresh DEA solutions but remain significantly lower than the saturated counterparts, further confirming partial CO_2_ desorption.

Gas chromatography analysis was conducted on the gaseous phase, and the results of duplicate samples of the desorbed gases of each campaign are presented in Table S6. This analysis revealed that the collected gas samples consisted of more than 99% CO_2_ with only trace amounts of oxygen, confirming the effectiveness of the desorption process. Although the presence of H_2_S was detected in the biogas stream of the second biogas plant, H_2_S was not detected in the gas stream after the desorption step, due to its concentration being below the detection limit of the instrument.

### 3.2. Biomass Combustion Plant Results

As a first step, elemental analysis of biomass feedstock was conducted to characterize its inorganic fraction (e.g., Cl, S, alkali and heavy metals), which is known to influence the formation of gas-phase impurities during thermal conversion and their presence in flue gases. This initial assessment informed and supported the sampling procedure and the interpretation of impurity presence along the value chain [46]. Following the experimental procedures described in Section 2.2, the quantified concentrations of the targeted impurities are reported below and discussed in relation to relevant process specifications. The associated measurement uncertainty of the analytical result was estimated to be approximately 5%. After completion of the sampling phase, the saturated DEA solutions with CO_2_ were transferred off-site and analyzed using accredited methodologies appropriate for each target compound. The analytical results are categorized into three groups: those reflecting the composition of the raw flue gas stream after the DeSOx/bagfilter and prior to CO_2_ capture, those representing the saturated amine solution with CO_2_ and those representing the composition of the recovered CO_2_ stream following the absorption process.

#### 3.2.1. Biomass Feedstock Elemental Analysis

A detailed physicochemical characterization of the biomass feedstock, including elemental and heavy metal analysis, is provided in Table 9 and Table 10.

The elemental and heavy metal analysis of the biomass feedstock confirmed the expected partitioning trends, which form the basis for the subsequent discussion. Trace elements during combustion are primarily governed by their thermodynamic stability, volatility and affinity for mineral phases. Elements with due volatility, such as Ca, Mg, Al, Fe, Ti, Mn, Ba, B, Cr, Ni, Co, Mo, Be and Y are predominantly retained in the bottom ash, because they form stable oxides with high melting points, promoting their retention in bottom ash [47]. In contrast, elements capable of forming volatile chlorides or oxides at combustion temperatures are the highly volatile species, including Hg, Se, Cl, and S. These species may vaporize and subsequently recondense onto fine fly ash particles during flue gas cooling [46]. Alkali metals, particularly potassium (K) and Sodium (Na), exhibit high mobility in combustion systems due to their low melting point compounds, yet their tendency to condense onto particulate matter enables effective downstream removal through filtration [46,48]. Meanwhile, elements like Zn, Pb, Cd, As, Sn, Cu and Sb exhibit partial volatility and may appear in the flue gas depending on combustion conditions [47]. The final gas-phase concentration of each element therefore reflects a dynamic balance between volatilization, homogenous gas-phase transport, heterogeneous condensation and particulate capture efficiency. To validate these assumptions and assess filter efficiency, selected low-volatility metals, V, Mn, Cr, Ti, Ni and Co, were monitored. These elements occur in moderate concentrations in biomass and are readily measurable in flue gas streams, while partially volatile and volatile species were included to ensure a comprehensive assessment of potential gas-phase carryover. The final list of target impurities included V, Mn, Cr, Ti, Ni, Co, Zn, Pb, Cd, As, Sn, Cu, Sb, Hg, Cl, and S, ensuring comprehensive evaluation of both particle-bound and volatile species.

#### 3.2.2. Pre-Capture Measurements

The first set of results corresponds to the composition of the untreated flue gas prior to its entry into the CO_2_ capture unit (Table 11). A preliminary assessment of the flue gas composition allows for the identification of impurities that may pose challenges within the value chain. Among these, hydrogen chloride appears to have the most significant impact due to its relatively high concentration, which measured 5.97 ppm in the gas stream. Zinc was also detected at a concentration of 0.0315 ppm, exceeding the established catalyst tolerance limit. Additionally, elevated levels of NO_x_ were detected, which could potentially affect not only the catalyst performance but also the MGA unit, since NO_x_ species are known to react with amine solvents, potentially reducing absorption efficiency and causing solvent degradation [49]. The reported concentrations are subject to an estimated measurement uncertainty in the range of 5–15%.

The experimental results confirmed the theoretical assumptions (Section 3.2.1), demonstrating that the selected trace elements (V, Mn, Ti, Cr, Ni and Co) were effectively retained by the particulate filtration system. The measured concentrations of those in the flue gas downstream of the filter were either negligible or below detection limits, indicating a high retention efficiency.

#### 3.2.3. Measurements in Saturated Amine Solutions

**Figure 10:**
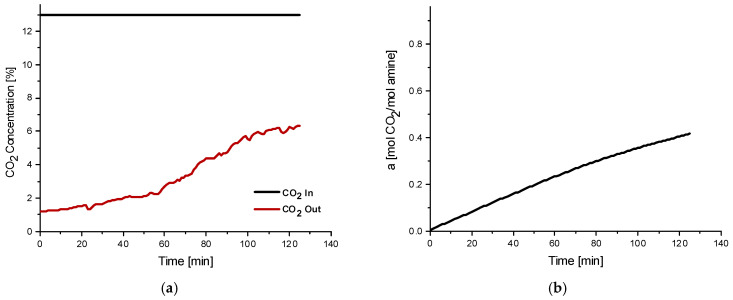
Results of the 2 h MGA campaign at the biomass combustion plant, (**a**) CO_2_ concentration (vol%) variation in the gaseous stream after MGA with time, and (**b**) absorption solvent CO_2_ loading (mol CO_2_/mol DEA).

The experimental results from the MGA campaign conducted at the biomass combustion plant are presented in Figure 10, illustrating the variation of CO_2_ concentration over time in the gas stream downstream of the MGA unit as well as the corresponding CO_2_ loading of the absorption solvent. Over the 2-h campaign, the CO_2_ concentration downstream of the MGA unit approached the initial upstream concentration, indicating the solvent saturation. Under the described experimental conditions, the CO_2_ loading capacity of the 2M DEA aqueous solution was observed to exceed 0.4 mol_CO_2__/mol_DEA_. It is anticipated that extending the absorption duration would result in the downstream concentration asymptotically approaching the upstream value, with a further increase in CO_2_ loading beyond 0.4 mol_CO_2__/mol_DEA_.

**Figure 11:**
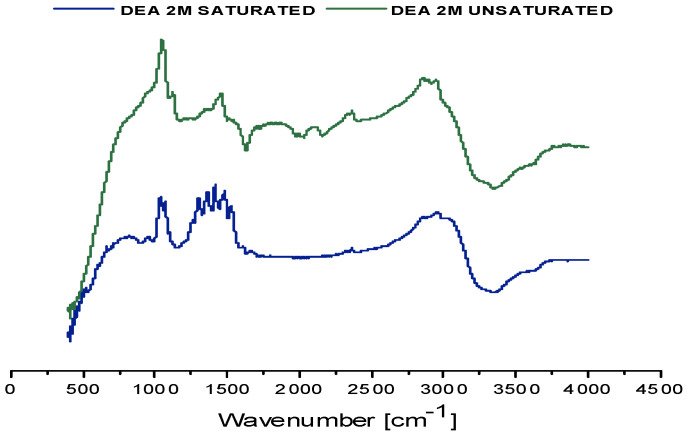
Comparison of the FT-IR spectra (cm^−1^) of saturated and unsaturated 2M DEA samples obtained at the MGA campaign in the biomass combustion plant.

Consistent with the previous campaigns, the density of the saturated 2M DEA solvent was measured at 25 °C. This value corresponds to an increase of 0.0357 g/mL compared to the density of the unsaturated 2 M DEA solution, reflecting the absorption of CO_2_. FT-IR spectroscopy was also performed on these samples (Figure 11). A detailed correspondence between the observed absorption bands and their associated chemical bonds is provided in Table 12.

Table 13 presents the quantities of trace contaminants captured through the amine absorption process. Partial blockage was observed in the gas inlet tubing connected to the membrane contactor unit. This phenomenon is attributed to localized condensation of residual water vapor, likely occurring in the unheated section of the tubing or downstream of the silica gel stage. At low ambient temperatures, residual moisture in the gas stream can undergo rapid condensation upon contact with surfaces below the dew point. This is especially likely if thermal insulation or heating capacity is insufficient to maintain adiabatic conditions [50]. Such condensation can result in transient flow obstruction, potentially compromising membrane efficiency and introducing uncertainties in gas-phase measurements [51]. Condensate samples were collected and retained for further compositional analysis, with focus on identifying inorganic species, especially Cl^−^. To quantify the chloride content, ion chromatography was employed; 1.64 ppm of chloride in the liquid condensate was detected. The results reflect a comprehensive assessment of impurity evolution across all stages, from the raw biomass to the treated CO_2_ stream. The observed reduction in contaminant levels demonstrates that the majority of impurities generated during biomass combustion can be removed through the implemented treatment sequence. The results show that the concentration of all trace components, including chlorides, which in previous campaigns appeared at high levels, has been substantially reduced after CO_2_ absorption. The degree of sequestration achieved is enough to bring impurity levels below the threshold specifications for catalyst toleration, thus reducing extensively major gas cleaning protection measures. Due to the interference caused by the significant nitrogen content originating from the DEA solvent, the quantification of NO_x_ was not feasible. Consequently, the future implementation of an on-site DeNO_x_ system is recommended to condition the gaseous stream.

**Figure 12:**
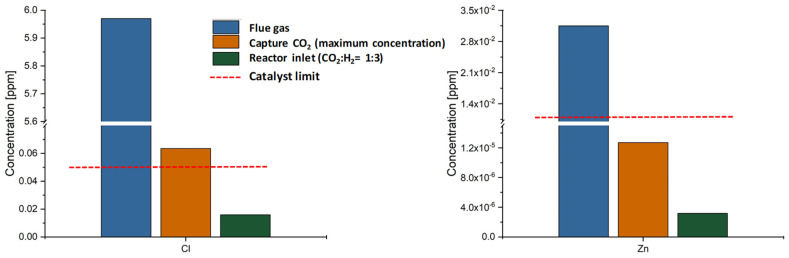
Concentration of Chloride and Zinc (ppm) at three key stages of the biomass combustion value chain. Trends show a 90% reduction achieved by the MGA unit; concentrations are compared against catalyst tolerance limit.

Figure 12 illustrates the concentration of Chloride and Zinc across the entire methanol production chain, which were the prevailing trace elements detected at concentrations in the flue gas, exceeding the tolerance thresholds of the methanol synthesis catalyst. In this case, the captured CO_2_ values refer to the theoretical maximum impurity concentration that could be contained in the recovered CO_2_ stream if it were completely recovered. The application of MGA achieved a reduction of approximately 99% for both Chlorides and Zinc; specifically, a reduction in chloride concentration from 5.97 ppm in the flue gas to 0.064 ppm in the CO_2_ stream intended for methanol synthesis. However, the Chloride concentration at the reactor inlet, measured at 0.016 ppm, is below the catalyst tolerance limit. This significant decrease suggests that the use of the MGA unit purifies the CO_2_ stream to a large extent and thus minimizes the need for extensive gas cleaning on-site and for methanol synthesis.

#### 3.2.4. Regeneration CO_2_

**Figure 13:**
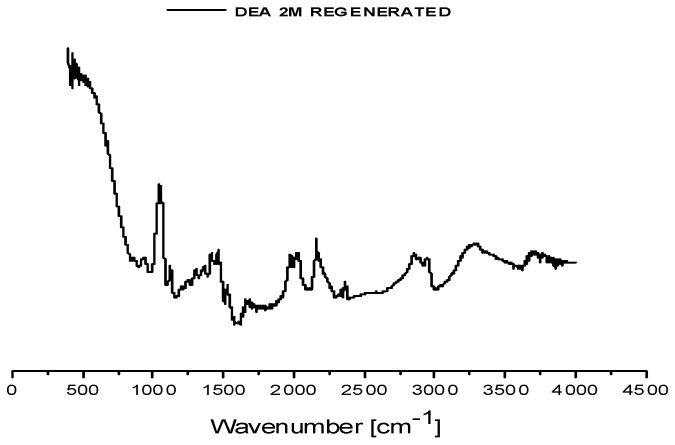
FT-IR spectra (cm^−1^) of the regenerated 2M DEA sample from the biomass combustion campaign.

The regeneration experiments for the saturated DEA solvent followed the same procedure and utilized the same experimental setup as in biogas campaigns, ensuring consistency in performance evaluation. The saturated DEA solution from the biomass combustion campaign was regenerated and analyzed to assess the extent of CO_2_ release and any remaining impurity content. The FT-IR spectrum of the regenerated sample displayed the same peak positions as in the saturated state, with reduced intensities, indicating partial removal of CO_2_-loaded species while preserving the chemical structure of the solvent (Figure 13). Density measurements supported this observation, showing a decrease from 1.0564 g/mL (saturated) to 1.0257 g/mL (regenerated), consistent with successful desorption.

Gas chromatography analysis of the desorbed gas confirmed high CO_2_ purity (>99.9 vol%), with the only trace oxygen detected and no measurable levels of sulfur compounds or hydrocarbons, validating the effectiveness of the regeneration process. Measurements were performed in duplicate, consistent with the biogas analyses, confirming the repeatability of the regeneration process. Consistent trends were observed across all campaigns, regardless of the CO_2_ source. While certain inlet streams contained higher levels of specific impurities (amount of chlorides, in biogas sampling campaigns), the regeneration step effectively minimized residual contamination in the recovered CO_2_ stream, confirming the robustness of the MGA process under varying feed conditions. Quantitative GC analysis of duplicate samples (Table 14) showed CO_2_ concentrations ranging from 99.812 to 99.959 vol%, with oxygen detected at trace levels (0.041–0.188 vol%). All other analyzed components, including H_2_, N_2_, CO, CH_4_, hydrocarbons (C_1_–C_5_), and H_2_S, were below the analytical detection limits of the instrument (0.1 vol% for permanent gases, 0.01 vol% for hydrocarbons, and 0.05 vol% for H_2_S). These results confirm that the thermal regeneration step produces a highly purified CO_2_ stream suitable for downstream utilization.

The primary risk identified prior to CO_2_ capture relates to elevated NO_x_ concentrations observed during biomass combustion, particularly NO_2,_ which may promote oxidative degradation of DEA, potentially forming nitrosamine-related species and contribute to the gradual buildup of non-regenerable salts during long-term operation and solvent loss [41]. In the present work, no direct quantification of solvent degradation products was performed, as the focus was on CO_2_ capture performance and impurity transfer to the recovered CO_2_ stream. However, FT-IR analysis of fresh, loaded, and regenerated solvent samples did not reveal additional absorption bands indicative of significant solvent degradation, suggesting that degradation effects were limited under the operating conditions applied. Nevertheless, long-term exposure to higher concentrations of acid gases could promote solvent degradation and heat-stable salt formation, which should be considered in future investigations.

Although primary gas treatment systems were implemented, sampling results indicate the necessity for a secondary purification step to meet quality standards. In particular, NO_x_ removal is essential to protect the environment, with selective catalytic reduction remaining among the most effective DeNO_x_ technologies [52]. Post-capture sampling showed that the CO_2_ stream consistently meets proposed ISO impurity limits (ISO27913:2024, ISO/TR27929), allowing for safe transportation without extensive on-site conditioning [13,14]. However, depending on the required phase state (e.g., liquid, dense or supercritical), further cleaning such as dehydration may be necessary to ensure operational safety and efficiency [53].

To provide an evidence-based framework for gas conditioning, a comprehensive decision-making table for gas has been included in Appendix A. This table maps the measured impurity concentrations and their associated uncertainties against the catalyst tolerance threshold and ISO transport specifications (ISO 27913:2024 and ISO/TR 27929) [13,14]. Based on these quantitative comparisons, specific conditioning sequences, ranging from standalone MGA to integrated DeNO_x_ and guard bed configurations, are recommended to ensure the long-term integrity of the methanol synthesis catalyst.

## 4. Conclusions

Trace impurities in biogenic CO_2_ streams significantly influence the integrity of the entire carbon utilization value chain, including capture, transportation and catalytic conversion to methanol. This study provided a holistic characterization of actual industrial bio-CO_2_ sources, systematically evaluating CO_2_ quality at three critical stages: prior to capture, post capture and pre-synthesis. To ensure the integrity of the data, a standardized multi-point sampling protocol was implemented under steady-state industrial operations. Specifically, isokinetic sampling was employed for biomass combustion flue gas to capture representative particulate and gaseous species, while in-series measurements were conducted at biogas facilities downstream of existing desulfurization units.

The field deployment of the modular membrane gas absorption (MGA) system demonstrated that it serves as a dual-purpose technology, achieving high capture rates while simultaneously acting as a primary purification stage. Specifically, the MGA unit achieved approximately 99% removal efficiency for critical contaminants such as Chlorides and Zinc in biomass combustion flue gas, bringing their concentrations (0.016 ppm at the reactor inlet) well within the stringent tolerance limits of commercial Cu/ZnO/Al_2_O_3_ catalysts. Furthermore, the recovered CO_2_ purity exceeded 99.9 vol% across all campaigns, validating the robustness of the solvent regeneration process.

However, the results also highlighted significant source-specific challenges. In biogas facilities, residual chloride concentrations of up to 9.8 ppm were detected post-MGA treatment, necessitating targeted secondary polishing steps, such as wet scrubbing or guard beds, to prevent irreversible catalyst poisoning. In biomass combustion, the presence of elevated NO_x_ levels (up to 65.9 ppm) poses a dual risk: potential catalyst deactivation and chemical degradation of the DEA solvent. Consequently, the integration of upstream DeNO_x_ units (e.g., SCR) is recommended to ensure the long-term stability of the capture system.

In conclusion, while biogenic CO_2_ is a viable feedstock for carbon-neutral marine fuels, no single cleaning method can effectively address all cases. A multi-stage, source-specific conditioning strategy, combining pre-treatment, MGA-based capture and dedicated polishing units, is essential to manage feedstock variability and meet transportation standards. Future research should address seasonal fluctuations, long-term catalyst exposure to residual trace impurities and comprehensive techno-economic and environmental assessments of the integrated bio-CO_2_-to-methanol value chain.

## Figures and Tables

**Table 1:** Technical characteristics of the investigated bio-CO_2_ facilities. Measurements were conducted under steady-state conditions at nominal capacity. Gas composition is expressed in vol%.

Facility ID	Type of Plant	Capacity/Power	Primary Feedstock	Gas Composition	Pre-Treatment Units	Sampling Point
Biogas Plant No.1	Anaerobic Digestion (CHP)	1 MW	Residues for beer, olive oil, and dairy	60 ± 2% CH_4_/36 ± 1% CO_2_	Drying and desulphurization	After desulphurization and before CHP unit
Biogas Plant No.2	Anaerobic Digestion (CHP)	950 kW	Fruit residues and dairy by-products	61 ± 2% CH_4_/37.5 ± 1% CO_2_	Desulphurization	
Biomass combustion plant	Combustion (Boiler)	2 × 15 MW	Agricultural residues	3–15% CO_2_	DeSO_x_ and bag filter	After bag filters and before flue gas fan

**Table 2:** Impinger solution parameters for flue gas impurity analysis in biomass combustion campaign. Sampling was performed using specific absorption solutions with a duration of 45–60 min and solution quantities in mL.

Impurity		Absorption Solution	Duration [min]	Number of Impingers	Solution Quantity [mL]
Halogens	HCl	H_2_O	60	5	100
	HF	NaOH 0.1M			
Heavy metals		3.3% HNO_3_/1.5% H_2_O_2_	60	4	100
Mercury		4% K_2_Cr_2_O_7_/20% HNO_3_	45	2	60

**Table 3:** Technical specifications of the 3M Liqui-Cel^TM^ 2.5 × 8 EXF membrane contactor. Parameters include effective membrane area (cm^2^), fiber dimensions (cm), pore size (μm) and fiber material used in the MGA capture campaigns [25,26].

Membrane Contactor	
Effective membrane area (cm^2^)	14,000
Cartridge configuration	Cross-flow
Fiber type	X40
Fiber ID/OD (cm)	0.024/0.03
Fiber Material	Polypropylene
Fiber porosity (%)	40
Fiber pore size (μm)	0.03
Number of fibers	10,000
Length (cm)	27.7
Diameter (cm)	7.7

**Table 4:** Experimental operating conditions during the 2 h MGA sampling campaigns. Specifications include pressure (bar), temperature (°C) and gas/liquid flow rates (L/min) for the two biogas plants and the biomass combustion facility.

Parameter		Pressure (bar_g_)		ΔΡ (bar_g_)	Temperature (°C)	Flow (L/m)
		Inlet	Outlet			
Biogas Plant No.1	Biogas (g)	0.15	0	0.15	22.0	16.0
	Absorbent (l)	0.4	0.2	0.2	20.0	2.5
Biogas Plant No.1	Biogas (g)	0.15	0	0.15	30.5	16.0
	Absorbent (l)	0.4	0.2	0.2	23.0	2.5
Biomass combustion plant	Flue gas (g)	0.07	0	0.07	22.5	9.7
	Absorbent (l)	0.35	0.15	0.2	22.5	2.65

**Table 5:** Average composition (vol% and ppm) of biogas streams. Analysis was performed using a Cubic-Ruiyi Gasboard-3200 online Infrared Biogas analyzer. Reported values reflect steady-state operation with measurement repeatability better than 1%.

Component	Biogas Plant No.1	Biogas Plant No.2
CH_4_	60.2%	61.7%
CO_2_	37.5%	38.4%
O_2_	0.32%	0.12%
H_2_S	0 ppm	20–30 ppm

**Table 6:** Correspondence between chemical bonds and FTIR spectrum peaks in the campaign in the biogas plants.

Bond	Unsaturated DEA 2M Wavelength (cm^−1^)	Saturated DEA 2M Biogas Wavelength (cm^−1^)	
		Biogas Plant No.1	Biogas Plant No.2
C-N	1126–1050	1070–1030, 1300	1060–1030, 1300
C-H_2_	1436, 1300	929–880, 1300, 1630–1326	940–860, 1300, 1650–1340
N-H	2200–2000, 1700–1600, 3000–2780	929–880, 1630–1326, 3000–2780	940–860, 1650–1340, 3000–2800, 2000
N-CH_2_	3000–2780	3000–2780	3000–2800
C-H	3000–2780	3000–2780	3000–2800
O-H	3600–3400	3600–3400	3600–3400
C-O	-	1300	1300
C=O	-	2300, 1630–1326	2300, 1630–1326

**Table 7:** Maximum potential concentration of trace impurities (ppm) in the recovered CO_2_ and methanol reactor inlet. Concentrations were determined assuming 100% recovery from the saturated DEA solvent. Analysis utilized IC, CVAAS and FAAS with an estimated analytical measurement uncertainty of 10–20%.

Parameters/Compounds	Content [ppm]				LOQ* [ppm]
	Biogas Plant No.1		Biogas Plant No.2		
	Per CO_2_ Absorbed	Reactor Inlet	Per CO_2_ Absorbed	Reactor Inlet	
Chlorides (Cl^−^)	16.5	4.1	39.3	9.8	0.004
Fluorides (F^−^)	9.95 × 10^−3^	2.49 × 10^−3^	5.37 × 10^−3^	1.34 × 10^−3^	7.89 × 10^−4^
SO_4_^2−^	b.t.**	b.t.**	b.t.**	b.t.**	b.t.**
Sulphides (S^2−^)	9.47 × 10^−4^	2.37 × 10^−4^	2.1 × 10^−4^	5.26 × 10^−5^	1.58 × 10^−4^
Arsenic (As)	n.d.***	n.d.***	n.d.***	n.d.***	3.95 × 10^−5^
Mercury (Hg)	n.d.***	n.d.***.	n.d.***	n.d.***	7.89 × 10^−6^
Antimony (Sb)	n.d.***	n.d.***	n.d.***	n.d.***	7.89 × 10^−5^

LOQ*: Limit of Quantification; b.t.**: below threshold; n.d.***: not detected.

**Table 8:** Density measurements in g/mL at 25 °C of fresh, saturated and regenerated solvent samples from the three demo campaigns. Measurements were obtained using a Digital Density Meter (ASTM-D42052-18a).

Sample	Fresh	Saturated	Regenerated
Biogas plant No.1	1.0207	1.0744	1.0251
Biogas plant No.2	1.0207	1.0719	1.0265

**Table 9:** Elemental analysis of the biomass feedstock (99% wood, 1% pellet). Results were obtained according to the EN15404:2011 standard.

Component	Unit	Value
Moisture	%	18.9
Crude ash		2.2
Nitrogen (N)	(%DM)	1.7
Sulfur (S)	ppm	228.2
Oxygen (O)	(%DM)	43.7
Hydrogen (H)		6.1
Carbon (C)		47.4
Chlorides (Cl)	mg/L	274.8

**Table 10:** Heavy metals analysis (ppm) of the biomass feedstock. Quantification of inorganic constituents was conducted using ICP-MS/XRF equipment.

Component	Concentration [ppm]	Component	Concentration [ppm]
Boron (Bo)	1.8	Yttrium (Y)	57.16
Sodium (Na)	39.5	Cobalt (Co)	1.5
Magnesium (Mg)	218.1	Nickel (Ni)	1.03
Aluminum (Al)	183.6	Zinc (Zn)	5.35
Potassium (K)	567.4	Arsenic (As)	0.082
Calcium (Ca)	1719.7	Selenium (Se)	<0.001
Chromium (Cr)	1.6	Cadmium (Cd)	0.028
Manganese (Mn)	10.2	Antimony (Sb)	0.019
Iron (Fe)	152.2	Barium (Ba)	5.05
Mercury (Hg)	0.108	Molybdenum (Mo)	0.056
Lead (Pb)	0.43	Tin (Sn)	<1.0
Beryllium (Be)	<0.06	Copper (Cu)	0.8
Titanium (Ti)	14.4	Vanadium (V)	0.34

**Table 11:** Impurity concentration profile (vol% and ppm) in the untreated flue gas stream. Gaseous species were measured using an ENVEA MIR 9000P portable gas analyzer, and heavy metals were quantified by ICP-AES. Reported values include estimated measurement uncertainty of 5–15%.

Content	Value
CO_2_ [vol%]	13.3
O_2_ [vol%]	7.73
CO [ppm]	124
NO_x_ [ppm]	65.9
SO_x_	<LOD (=0.27)
Impurities	Value [ppm]
HCl	5.97
HF	Below threshold (<0.05)
Cadmium (Cd)	0.0001
Titanium (TI)	Below Threshold (<0.004)
Arsenic (As)	Below Threshold (<0.0026)
Chromium (Cr)	0.0111
Cobalt (Co)	Below Threshold (<0.0014)
Copper (Cu)	0.0014
Manganese (Mn)	0.0062
Nickel (Ni)	0.0072
Antimony (Sb)	Below Threshold (<0.0016)
Vanadium (V)	Below Threshold (<0.0038)
Lead (Pb)	0.0026
Zinc (Zn)	0.0315
Tin (Sn)	Below Threshold (<0.0016)
Mercury (Hg)	0.00016

**Table 12:** Correspondence between chemical bonds and FTIR spectrum peaks in the campaign in the biomass combustion plant.

Bond	Unsaturated DEA 2M Wavelength (cm^−1^)	Saturated DEA 2M Wavelength (cm^−1^)
C-N	1126–1050	1090–1040
C-H_2_	1436, 1300	1715–1620, 1536–1250, 950–840
N-H	2200–2000, 1700–1600, 3000–2780	3600–3420, 3080–2836, 1715–1620, 1536–1250, 950–840
N-CH_2_	3000–2780	3080–2836
C-H	3000–2780	3600–3420, 3080–2836
O-H	3600–3400	3600–3420, 3080–2836
C-O	-	1536–1250
C=O	-	2363, 1715–1620, 1536–1250

**Table 13:** Trace contaminants (ppm) captured in the 2M DEA solution during the biomass combustion campaign. Analysis was performed via Ion chromatography (CEN/TS 15289) and ICP-MS. Values represent conversion upper-bound estimations for the reactor inlet assuming an ideal 1:3 CO_2_:H_2_ ratio.

Parameters/Compounds	Analytical Method	LOQ [ppm]	Per CO_2_ Absorbed [ppm]	Reactor Inlet [ppm]
Chlorides (Cl^−^)	CEN/TS 15289 (ic) mod.	0.06	0.06353	0.01588
Fluorides (F^−^)		0.03	n.d.*	n.d.*
Sulphides (S^2−^)		0.16	n.d.*	n.d.*
Cadmium (Cd)	Microwave digestion ICPMS	0.0002	n.d.*	n.d.*
Mercury (Hg)		3 × 10^−5^	0.00005	0.00001
Chromium (Cr)			n.d.*	n.d.*
Copper (Cu)		0.0002	n.d.*	n.d.*
Manganese (Mn)		0.0002	0.0003	0.00008
Nickel (Ni)		0.0002	n.d.*	n.d.*
Lead (Pb)		0.0002	n.d.*	n.d.*
Zinc (Zn)		0.0005	0.00001	≈0

n.d.* = not detected.

**Table 14:** Gas chromatographic composition of duplicate samples of desorbed gas collected during each demonstration campaign.

Normalized GC Analysis		Biogas Plant No.1		Biogas Plant No.2	
CO_2_	vol%	99.864	99.812	99.959	99.916
H_2_	vol%	0.000	0.000	0.000	0.000
O_2_	vol%	0.136	0.188	0.041	0.084
N_2_	vol%	0.000	0.000	0.000	0.000
CH_4_	vol%	0.000	0.000	0.000	0.000
CO	vol%	0.000	0.000	0.000	0.000
H_2_S	vol%	0.000	0.000	0.000	0.000
Total	vol%	100.00	100.00	100.00	100.00

## Data Availability

Data is contained within the article. Raw data are available on request from the corresponding author and will be curated according to the Data Management Plan applicable to the M^2^ARE project Grant Agreement No. 101136080.

## Supplementary Materials

The following supporting information can be downloaded at: https://www.mdpi.com/article/10.3390/membranes16030106/s1, Table S1: Standards for analysis of the saturated amine solution; Table S2: Standards for the measurement of impurities and for the analysis of the spent absorption solutions in the flue gas stream from the combustion plant; Table S3: Technical characteristics of the Cubic-Ruiyi Gasboard–3200 Online Infrared Biogas Analyzer; Table S4: Detected Siloxane species and their limited concentration in biogas streams in the examined biogas plants; Table S5: Density measurements for the saturated and unsaturated DEA solutions; Table S6: Gas chromatography analysis of duplicate samples of the desorbed gases of each demonstration campaign; Table S7: Normalized gas chromatography analysis of duplicate samples of the desorbed gases of each demonstration campaign.

## Abbreviations

The following abbreviations are used in this manuscript:

ASTM	American Society for Testing and Materials
CHP	Combined Heat and Power
CVASS	Cold Vapor Atomic Absorption Spectroscopy
DEA	Diethanolamine
DeNO_x_	Denitrification
EN	European Norm
FID	Flame Ionization Detection
FT-IR	Fourier Transform Infrared
G/L	Gas to Liquid
ICP-MS/XRF	Inductively Coupled Plasma-Mass Spectrometer/X-Ray Fluorescence
IMO	International Maritime Organization
ISO	International Organization For Standardization
MGA	Membrane Gas Absorption
MeOH	Methanol
SMEWW	Standard Methods for the Examination of Water and Wastewater
TCD	Thermal Conductivity Detector
T_pr_	Temperature of heated probe
T_f_	Temperature of heated filter

## Appendix A

**Table A1:** Comprehensive summary of measured impurity concentrations in the CO_2_ stream, compared against transportation and catalyst protection limits based on [13,14,15,52,54,55,56].

Impurity	Value [ppm]	Transportation [ppm]			Catalyst [ppm]	Additional Conditioning (After MGA)	Rationale
		Pipeline		Ships/Trucks/ Rails			
		Dense	Supercritical				
Biogas Plant No.1							
(Cl^−^)	4.1	n.m.*			0.05	MGA + Guard bed/Wet scrubber	Required to prevent irreversible catalyst poisoning
(F^−^)	2.49 × 10^−3^				0.05	MGA only	MGA acts as the primary capture and purification process
(S^2−^)	2.37 × 10^−4^	55	5	9	0.05		
SO_4_^2−^	b.t.***	1	10	10	0.05		
(As)	n.d.**	n.m.*			0.005		
(Hg)	n.d.**	5	-	0.03	0.005		
(Sb)	n.d.**	n.m.*			0.005		
Biogas Plant No.2							
(Cl^−^)	9.8	n.m.*			0.05	MGA + Guard bed/Wet scrubber	Required to prevent irreversible catalyst poisoning
(F^−^)	1.34 × 10^−3^				0.05	MGA only	MGA acts as the primary capture and purification process
(S^2−^)	5.26 × 10^−5^	55	5	9	0.05		
SO_4_^2−^	b.t.***	1	10	10	0.05		
(As)	n.d.**	n.m.*			0.005		
(Hg)	n.d.**	5	-	0.03	0.005		
(Sb)	n.d.**	n.m.*			0.005		
Biomass combustion plant							
(Cl^−^)	0.01588	n.m.*			0.05	MGA only	MGA acts as the primary capture and purification process
(F^−^)	n.d.**				0.05		
(S^2−^)	n.d.**	55	5	9	0.05		
(Cd)	n.d.**	n.m.* n.m.*			0.005 0.005		
(TI)	n.d.**						
(As)	n.d.**						
(Cr)	n.d.**						
(Co)	n.d.**						
(Cu)	n.d.**						
(Mn)	7.54 × 10^−5^						
(Ni)	n.d.**						
(Sb)	n.d.**						
(V)	n.d.**						
(Pb)	n.d.**						
(Zn)	3.18 × 10^−6^						
(Sn)	n.d.**						
(Hg)	1.15 × 10^−5^	5	-	0.03			
NOx	65.9	96	50	-	1	DeNO_x_(SCR) + MGA	Protects both the MGA solvent and the synthesis catalyst

n.m.* = not mentioned; n.d.** = not detected; b.t.*** = below threshold.
