# Supplementary material for: Capture, Sampling and Analysis of Biogenic CO2 Streams for Methanol Synthesis
Source: Membranes (Basel). 2026 Mar 17;16(3):106. doi: 10.3390/membranes16030106 (PMC13028185; doi:10.3390/membranes16030106)
Supplement: Supplementary file 1 [file membranes-16-00106-s001.zip › membranes-4177148-supplementary.pdf]

Supplementary material (S.I)

Capture, sampling and analysis of biogenic CO_2 streams for methanol synthesis

E. Koliamitra, V. Mitrousis, Tz. Kraia, G. Kardaras, N. Lazaridou, T. Grekou, K. Fotiadis, D. Koutsonikolas, A. Asimakopoulou, M. Bampaou, K. D. Panopoulos

Chemical Process and Energy Resources Institute (CPERI), Centre for Research and Technology Hellas (CERTH), 57001 Thessaloniki, Greece

1. Bio-CO_2 facilities overview

Two biogas facilities, located in Northern Greece, were examined. Biogas plant No.1, is a 1-MW combined heat and power facility that primarily uses liquid cow manure as feedstock. The plant is designed for a nominal biogas production of 12,000 m^3 per day, with maximum capacity up to 19,000m^3. Depending on the season, residues for beer, olive oil, and dairy processing are also introduced, reflecting the variability commonly observed in agricultural regions. Biogas is produced in two sequential digesters, with most of the volume being generated in the first 30 days of operation at 44° C. The final gas composition remains stable at approximately 60±2% CH_4 and 36±1% CO_2. After drying and desulphurization, the biogas is used for electricity and heat generation, while the digestant is pasteurized and applied as fertilizer. Biogas plant No.2 with a capacity of 950 kW, also exhibits seasonal flexibility, processing fruit residues during harvest months and dairy by-products in other periods. Despite these shifts, the facility maintains a consistent gas composition (61±2% CH_4, 37.5±1% CO_2) and delivers up to 18,000 m^3/day of biogas. Beyond energy production, it contributes to sustainable agriculture by providing farmers with liquid organic by-products as soil enhancers.

The biomass CHP plant is connected to a large-scale district heating network in Northern Greece, supplying thermal energy to approximately 2,000 public and residential buildings. The combustion plant is equipped with two water tube boilers, each with a nominal thermal capacity of 15 MW. These boilers combust biomass, mainly agricultural residues, to produce superheated water at temperatures above 130 °C, which then circulates the district heating system. Operating at an efficiency of over 89%, the plant incorporates advanced emission control technologies, including flue gas cleaning systems ($DeSO_x$) and ash removal systems. In addition, it features automated operation control, continuous monitoring systems and analytical devices for measuring critical parameters such as exhaust gas composition. The plant has the capacity to operate on different feedstock sources such as agricultural residues collected from local farmers but also solid recovered fuel derived from the processing of non-hazardous municipal solid waste.

2. Sampling in biogas plants

The standard used for the evaluation of the saturate amine solution is presented in Table S1. These methods are also used for the analysis of the saturated amine solution of biomass combustion plant.

Table S1: Standards for analysis of the saturated amine solution.

Parameters	Analytic Methods	Reference standard
**Chlorides (Cl^-)** **Fluorides (F^-)**	Ion chromatography (IC)	SMEWW-4500

**SO_4^{2-}**		
**Sulphides (S^{2--})**	Spectrophotometry (UV-VIS)	ISO 10530:2002
**Arsenic (As)**	Atomic Absorption Spectometry (AAS)	ASTM D2972-03
**Mercury (Hg)**	Cold Vapor Atomic Absorption Spectometry (CVAAS)	ASTM D3223:2000
**Antimony (Sb)**	Flame Atomic Absorption Spectroscopy (FAAS)	ISO 15586:2003

3. Sampling in biomass combustion plant

Table S2 presents the reference standards and analytic methods used for the measurement and quantification of impurities in the flue gas stream, including the corresponding methods applied to the analysis of spent absorption solutions.

Table S2: Standards for the measurement of impurities and for the analysis of the spent absorption solutions in the flue gas stream from the combustion plant.

**Component**	**Reference Standards**		**Analytic Method for quantification**
	**Measurement**	**Quantification**	
**HCl**	EN-1911-1	ISO15713	Ion Chromatography (IC)
**HF**	ISO 15713	EN 14791	
**SO_2**	ISO 7935	EN ISO10304	
**O_2**	EN 14789	EN 14789	Paramagnetic Method
**CO_2**	ISO 12039	ISO 12039	Non-Dispersive Infrared (NDIR)
**CO**	EN 15058	EN 15058	
**NO_x**	EN 14792	EN 14792	Chemiluminescence Detection
**Hg**	EN 13211	EN ISO 17852<br>EN ISO13211<br>ISO 12846	Fluorescence Spectometry after oxidative digestion
**Heavy metals (As, Cd, Cr, Co, Cu, Mn, Ni, Pb, Sb, Tl, V, Zn, Sn)**	EN 14385	EPA Method 200.7<br>EN ISO 11885,<br>EN 13211,<br>EN 14385,<br>EN 14902	Inductively Coupled Plasma Atomic Emission Spectometry (ICP-AEs)

4. MGA unit specifications

The below table presents the technical characteristic of analyzer used to samplings.

Table S3: Technical characteristics of the Cubic-Ruiyi Gasboard–3200 Online Infrared Biogas Analyzer.

**Specifications**	**Gasboard – 3200 Online Infrared Biogas Analyzer**
**Measuring Components**	O_2, CO_2, CH_4, H_2S
**Measurement Range**	O_2 : (0 – 25)%

	CO_2 : (0 – 100) %<br>CH_4 : (0 – 100) %<br>H_2S : (0-1000) ppm
**Accuracy**	CO_2, CH_4 : ≤1% FS<br>O_2 ,H_2S : ≤2% FS
**Measurement Precision**	CO_2, CH_4, O_2 : 0.01%<br>H_2S : 1 ppm
**Repeatability**	≤ 1 %
**Response Time**	< 10 s
**Flow Rate**	0.7 – 1.2 lpm

5. Results

5.1 Extended results of biogas sampling campaigns

This section include results details on the analytical methodologies employed throughout the study. It presents the results of the siloxane and density measurements, general assignments of the FTIR spectral peaks relevant to the system, and supporting results from CO_2 regeneration experiments.

Table S4: Detected Siloxane species and their limited concentration in biogas streams in the examined biogas plants.

**Siloxane species**	**Results**		**Threshold [ppmv]**	**Method**
	**Biogas plant No.1**	**Biogas plant No.2**		
Tetramethylsilan	Below Threshold	Below Threshold	0.02771	VDI 2100 BI.2 (2010-11)
Trimethylsilanol	Below Threshold	Below Threshold	0.02771	VDI 2100 BI.2 (2010-11)
Hexamethyldisiloxan	Below Threshold	Below Threshold	0.01506	VDI 2100 BI.2 (2010-11)
Hexamethylcyclotrisiloxan	Below Threshold	Below Threshold	0.01099	VDI 2100 BI.2 (2010-11)
Octamethyltrisiloxan (L3)	Below Threshold	Below Threshold	0.01034	VDI 2100 BI.2 (2010-11)
Octamethylcyclotetrasiloxan	Below Threshold	Below Threshold	0.00824	VDI 2100 BI.2 (2010-11)
Decamethyltetrsiloxan (L4)	Below Threshold	Below Threshold	0.00824	VDI 2100 BI.2 (2010-11)
Decamethylcyclopentasiloxan	Below Threshold	Below Threshold	0.00659	VDI 2100 BI.2 (2010-11)
Total Silicon (calc.)	Below Threshold	Below Threshold	0.00379	VDI 2100 BI.2 (2010-11)

Sum of silicon organic compounds	Below Threshold	Below Threshold	0.00139	VDI 2100 BI.2 (2010-11)
Sum SiO_2	Below Threshold	Below Threshold	0.0407	VDI 2100 BI.2 (2010-11)

Table S5: Density measurements for the saturated and unsaturated DEA solutions.

Sample	Biogas plant No.1	Biogas plant No.2
**Unsaturated DEA [g/ml]**	1.0207	1.027
**Saturated DEA [g/ml]**	1.0744	1.0719

Table S6: Gas chromatography analysis of duplicate samples of the desorbed gases of each demonstration campaign.

Sample name<br>Normalized GC analysis		Biogas plant No.1		Biogas plant No.2	
**CO_2**	vol%	99.864	99.812	99.959	99.916
**H_2**		0.000	0.000	0.000	0.000
**O_2**		0.136	0.188	0.041	0.084
**N_2**		0.000	0.000	0.000	0.000
**CH_4**		0.000	0.000	0.000	0.000
**CO**		0.000	0.000	0.000	0.000
**H_2S**		0.000	0.000	0.000	0.000
**Total**		100.00	100.00	100.00	100.00

5.2 Extended results of biomass combustion campaigns

Results of the density measurements of the absorption solvent as well as supplementary results from the CO_2 regeneration experiments are presented below.

- **Unsaturated 2M DEA**: 1.0207 g/mL
- **Saturated 2M** DEA: 1.0564 g/mL

Table S2: Normalized gas chromatography analysis of duplicate samples of the desorbed gases of each demonstration campaign.

Component		Composition, vol%	
**CO_2**	vol%	99.148	99.908
**H_2**		0.000	0.000
**O_2**		0.852	0.092
**N_2**		0.000	0.000
**CH_4**		0.000	0.000

**CO**	0.000	0.000
**H_2S**	0.000	0.000
**Total**	**100.00**	**100.00**
